# ED-FNN: A New Deep Learning Algorithm to Detect Percentage of the Gait Cycle for Powered Prostheses

**DOI:** 10.3390/s18072389

**Published:** 2018-07-23

**Authors:** Huong Thi Thu Vu, Felipe Gomez, Pierre Cherelle, Dirk Lefeber, Ann Nowé, Bram Vanderborght

**Affiliations:** Robotics & MultiBody Mechanics Research Group (R& MM) and Artificial Intelligence Lab, Vrije Universiteit Brussel and Flanders Make; Pleinlaan 2, 1050 Brussel, Belgium; felipe.gomez.marulanda@vub.ac.be (F.G.); pierre.cherelle@vub.ac.be (P.C.); dlefeber@vub.ac.be (D.L.); ann.nowe@vub.ac.be (A.N.); Bram.Vanderborght@vub.be (B.V.)

**Keywords:** gait phase prediction, gait event detection, lower limb prosthesis, exoskeleton, gait recognition

## Abstract

Throughout the last decade, a whole new generation of powered transtibial prostheses and exoskeletons has been developed. However, these technologies are limited by a gait phase detection which controls the wearable device as a function of the activities of the wearer. Consequently, gait phase detection is considered to be of great importance, as achieving high detection accuracy will produce a more precise, stable, and safe rehabilitation device. In this paper, we propose a novel gait percent detection algorithm that can predict a full gait cycle discretised within a 1% interval. We called this algorithm an exponentially delayed fully connected neural network (ED-FNN). A dataset was obtained from seven healthy subjects that performed daily walking activities on the flat ground and a 15-degree slope. The signals were taken from only one inertial measurement unit (IMU) attached to the lower shank. The dataset was divided into training and validation datasets for every subject, and the mean square error (MSE) error between the model prediction and the real percentage of the gait was computed. An average MSE of 0.00522 was obtained for every subject in both training and validation sets, and an average MSE of 0.006 for the training set and 0.0116 for the validation set was obtained when combining all subjects’ signals together. Although our experiments were conducted in an offline setting, due to the forecasting capabilities of the ED-FNN, our system provides an opportunity to eliminate detection delays for real-time applications.

## 1. Introduction

**Figure 1:**
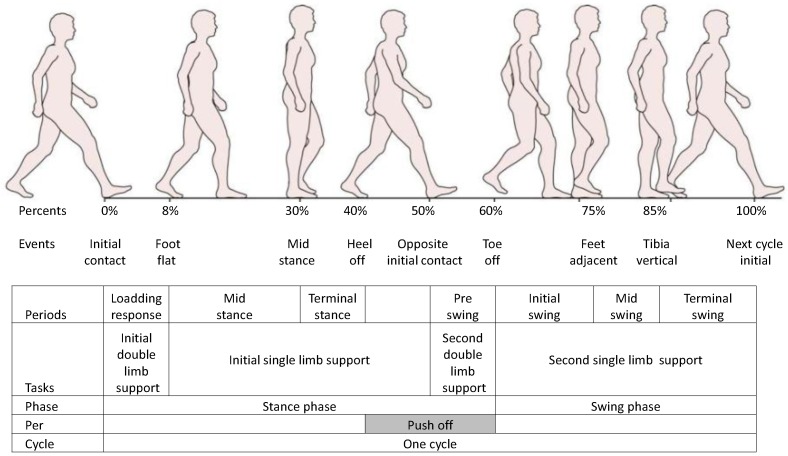
A gait cycle is described as a dynamic and continuous occurrence of eight phases from the heel-contact at 0% to the next heel-contact at 100% percent of the gait cycle. Phase 0 is initial double-limb support, which appears during the first 10% of the cycle. Phase 1 is mid-stance, which appears from 10% to approximately 30% of the gait cycle. The following 10% of the gait cycle is terminal-stance. The propulsion phase or toe-off occurs after foot flat from 40% of the gait. This stage pushes the body forwards and prepares for swing phase from approximately 60% of the gait cycle. Single-limb support occurs from foot flat until 50% of the gait-related opposite initial contact limb, typically at 50% of the gait cycle. The second double-limb support occurs from the opposite limb at 50% until the toe leaves the ground at 60% of the gait cycle. Then, the second single-limb support completes the cycle. The following phases are early swing at approximately 60% to 75% of the gait cycle, mid swing at approximately 75% to 85% of the gait cycle, and late swing at approximately 85% to 100% of the gait cycle. Adapted from [36].

Gait phase detection is a non-trivial problem for the new generation of powered prostheses and exoskeletons that are under development [1]. Gait phase detection algorithms are used to create and improve control strategies that permit prosthetic devices such as those presented in References [2,3,4,5] to work with more precision, safety, and stability. The objective of gait event detection algorithms is to detect non-delayed events in order to build control strategies for improving gait movement. For example, authors in [2] focused on a treatment to fix the amputee’s foot in the lifted position by an orthosis. The technology known as functional electrical stimulation (FES) facilitates the artificial generation of action potentials in subcutaneous efferent nerves during the swing phase of the paretic foot by applying tiny electrical pulses via skin electrodes or implanted electrodes. By modulating the frequency or dimensions of these pulses, one can control the contraction of paretic muscles and induce movements in the affected limbs based on gait phase transitions. The foot pitch and roll angles are assessed in real-time by means of an inertial measurement unit (IMU). They detected four phases based on measuring the angular velocity and accelerometer to control the pitch and roll of the foot. Another example is the Ankle Mimicking Prosthetic Foot (AMP-Foot) [4]. This device greatly relies on the accuracy of a gait event detection algorithm to precisely control the torque of the motor in the device. This device stores energy in the springs during the first event of gait from initial-contact (IC) to foot-flat (FF), then releases the energy stored in the push-off (PO) spring and transmits it to the ankle joint by controlling the direct-current (DC) of the motor at the moment of heel-off (HO). This joint effort provides a peak torque and power output to the amputee, producing a toe-off (TO) event. After the TO, the amputee enters a swing phase where the torque of the motor is returned to zero magnitude, allowing the foot to go back to its initial position, resulting in an IC event. Knowing when all these events take place allows the device to take action at the right moment. Due to the different gait terminologies used in different articles, in this paper we follow the wording of Figure 1. In this figure, a *gait cycle percent* is defined as a sample from the continuous space of the gait cycle, an *event* is viewed as a discrete representation of the percentage space, often labelled as IC, FF, HO and TO; a *gait period* is considered as an interval between events, and a *phase* is considered as a union of several periods that represent different stages of the gait cycle. Lastly, a full gait cycle is composed of a *stance phase* and a *swing phase*. A more detailed description of different gait events is provided in Section 3. In the gait percent detection literature, a large set of techniques for improving the performance of event and phase detection can be found. These include threshold-based methods [3,6,7,8], time-frequency analysis [9,10], peak heuristic algorithms [9,11,12], machine learning (ML) models [13,14,15,16,17,18,19,20,21], and combinations of these [22]. ML algorithms are among the most popular techniques to detect phases in off-line data (i.e., stored data) and for real-time data (i.e., data gathered in real time). For instance, authors in References [17,19] detected four event-phases using hidden Markov models (HMMs). Evans and Arvind [23] increased the number of event-phases to five, and applied a hybrid method that combined fully connected neural networks (FNNs) and HMMs. The model accuracy of these algorithms is dependent on the type of sensors used to gather the gait event signals. Currently, wearable sensors are widely used for gait phase recognition systems: wearable sensors such as foot switches [14,24,25], foot pressure insoles [6,16,26,27], electromyography (EMG) [28,29], IMUs [3,8,9,15,30,31,32,33,34], and joint angular sensors [20,21] are used specifically for gait detection. A review in [35] showed that foot switches and foot pressure insoles yield the highest accuracy for gait phase detection algorithms. However, these sensors are very sensitive to the placement of the insole, which can influence the accuracy and reliability of the model. Additionally, they have a short lifespan, as they are often exposed to shock forces of the gait. Consequently, foot switches and foot pressure sensors are not considered suitable for daily activity applications. In contrast, Joshi et al. used EMG sensors to accurately extract up to eight gait phases [29]. EMGs are sensors that measure specific muscle activities occurring during a task. Regardless of the amount of information that can be extracted from the EMG signals, a heavy pre-processing step (e.g., a complex combination of filters) is required before it can be directly used in a learning algorithm. Furthermore, these sensors are susceptible to artifacts generated by moisture that builds between the skin and the sensors, and to the way in which they are placed on the skin of the subject. Recently, IMUs including gyroscopes, accelerometers, and magnetometers have become more popular, as they are not affected by most of the limitations of the aforementioned sensors. IMUs are low-cost, low-energy, durable, and can be easily mounted on different parts of the human body. Moreover, a human walking gait is a periodic cycle where IMUs can measure the angular velocities and accelerations of the walking gait. As a result, these signals are composed of rich information that can be used to accurately predict the gait events. Similar to EMG signals, IMU signals are very sensitive to movement artifacts. This means that in some cases these signals may require a strong pre-processing step before they can be directly used for learning. We hypothesise that deep learning algorithms are best suited for gait phase detection using IMU signals, as they perform well on signals that have a medium-low signal-to-noise ratio.

This paper introduces a novel gait percent detection model based on deep learning (DL) algorithms that can predict a full gait cycle discretised within a 1% interval. Currently, most studies can accurately detect four to eight phases. However, for real-world applications, it may be not sufficient for controlling active prosthetic devices. As a result, a more densely sampled gait phase is needed in order to obtain more important gait information and give more possibilities of control. The purpose of this study is to open an opportunity for future active devices such as below-the-knee prosthetics to take full control of the gait by accurately predicting the gait percentage in densely sampled phases.

## 2. Related Work

Due to the capacity of IMU sensors to measure the velocities and accelerations of motion, they are generally used in the fields of gait phase detection, gait event detection, and gait detection. In this section, we analyse and evaluate the performances and time delays of several recent gait phase detection systems in the fields of transtibial prostheses and exoskeletons.

Evans and Arvind [23] presented a method for the detection of five gait phases based on a feed-forward neural network (FNN) embedded in the hidden Markov model (HMM) model. However, their sensor system had to use seven IMUs mounted on the foot, shank, both sides of the thigh, and one on the pelvis. An implementation of complex threshold rules was further applied in exoskeletons in References [8,37]. The study in [8] could detect seven gait phases, and Boutaayamou et al. could detect four events in their study, with a temporal accuracy of around 10 ms [37]. However, systems in [8,37] were required to use four sensors which were attached to the leg segments.

Several papers have proposed applications for robotic prostheses that use signals from one IMU attached to the shank or the foot [12,17,38,39] to detect four phases or events. Mannini et al. [17] and Muller et al. [39] proposed two different models to detect four gait events in real-time using one IMU. The former proposed an HMM and the latter proposed *afinite state automaton* to model the transitions between phases. Nevertheless, both cases showed a time delay limitation in on-line detection. For example, authors in [17] presented an average error latency of 62±47 ms for FS and 86±61 for HO. Moreover, authors in [39] reported delays when subjects were wearing or not wearing shoes. For example, they reported approximately 0.1±0.05 s for the TO and 0.01±0.07 s for the IC. Similar to our work, the recent study of Quintero et al. [40] worked on estimating the continuous progression of the gait cycle by extracting the relationship between the thigh angle and velocity extracted from one IMU. They transformed the angle–velocity relationship to polar coordinates in order to predict the gait percentage. However, a comparison between their results and ours would be unfeasible, as they only visually reported the accuracy of their technique. A study in [12] recently announced an effective algorithm that detects four gait events (e.g, IC, TO, mid-swing (MSw) and mid-stance (MSt)) based on a set of heuristic rules using one gyroscope attached to the shank of subjects performing activities of daily living such as normal walking, fast walking, ramp ascending, and ramp descending. However, this algorithm is limited to an off-line setting and to a non-detection of the push-off event, which is considered to be an important phase before toe-off. Although they state that their algorithm also works in an on-line setting, they do not show any evidence that supports their results. In summary, in the course of our literature review, we encountered gait event detection systems that limit their experiments to one IMU and to gait phases that were partitioned between two [9,41,42] and three phases [3]. In this limited framework, they illustrated that a high accuracy can be achieved. For instance, authors in [41,42] illustrated an accuracy of 100% in IC and TO event detection. Zhou et al. [9] showed an accuracy of above 98% for IC event detection and 95% for TO event detection on three different terrains. We also observed that in cases where the number of phases is increased (i.e., to four phases [12,15,17]), the gait phase detection performance also decreases. For example, the experiment in [15] yielded an average detection accuracy under 95% in every phase, while Mannini et al. [17] showed a long delay of detection from 45 ms to 100 ms. The experiment in [12] showed a mean difference error between the reference and the proposed system of approximately +4 ms for IC and −6.5 ms for TO.

Recently, most studies have focused on improving the detection accuracy with the purpose of applying real-time safety walking for amputees while at the same time increasing the number of detectable gait phases for better control of the robotic device. However, we previously saw that increasing the granularity of the phases leads to a significant decrease in the prediction accuracy. To cope with this trade-off, we built a new deep learning architecture called the exponentially delayed fully connected neural network (ED-FNN). This network is made to overcome most of the current limitations in gait phase detection algorithms, such as predicting gait events with one IMU attached to the lower shank with high phase granularity and no detection delay.

## 3. Materials and Methods

This section discusses the algorithms and materials that were used for our experiments section. First, the concept of *gait classification* is introduced. Second, we describe how the walking gait is typically discretised. Finally, a description of the ED-FNN architecture is provided.

### 3.1. The Division of the Human Walking Gait

The human walking gait is defined as a periodic cycle involving two legs from the initial contact of one foot on the ground to the following occurrence of the heel of the same event with the same foot. Typically, one gait cycle is divided into two main phases, including a stance phase which is approximately 60% of a gait cycle and a swing phase which is approximately 40% of the remaining gait cycle [43]. The heel-contact and the toe-off events mark the beginning of the stance phase and the swing phase, respectively.

The granularity of one step is equally divided into two, three, four, five, six, seven, and eight gait periods depending on the specific type of application. This is done to define the label of the different periods of the gait cycle [35]. An example of the different periods is shown in Figure 1, which illustrates eight periods that were summarised into one gait cycle based on 100 percent of the gait [35,43].

In our study, we classified a full gait cycle discretised within a 1% interval based on real-time measurements of the gait cycle. Doing so will allow prosthetic devices to have a wider spectrum of control in the cycle. For example, the prosthesis AMP-Foot 3 plus [4] stores energy in the springs at the mid-stance period and starts to inject positive energy at the terminal stance period by using a DC motor. The detection of the terminal stance period is important for injecting energy at the right time. At the initial swing and mid-swing periods, the trajectory for the next work situation (i.e., the next walking phase) needs to be set. It is of great importance to command the control of the prosthesis just before gait events happen in order to avoid action delay on the device. Therefore, an algorithm for detecting 100% of the gait is required for the next concept of gait phase detection. For applications that do not require a granular gait cycle, the phases can be mapped to the fundamentals of human gait phases (Table 1) in order to control the prosthesis when needed.

According to the fundamentals of human gait phases, the stance phase begins with IC from 0% to 10% of the gait cycle. Initial double-limb support appears during the first 10% of the gait cycle. The foot flat occurs from 10% until the heel leaves the ground at 40% of the gait cycle. Mid-stance appears at approximately 30% of the gait. Single-limb support occurs from foot flat until 50% of the opposite initial contact which is approximately at 50% of the gait cycle. The second double-limb support occurs from the opposite limb at 50% until the toe leaves the ground at 60% of the gait cycle. Then the second single-limb support starts until the cycle is complete. The following periods are early swing at approximately 60–75% of the gait cycle, mid-swing at approximately (75–85% of the gait cycle, and late swing at approximately 85–100% of the gait cycle. The fundamentals of human gait phases are shown in Figure 1.

### 3.2. Percent Segmentation Method for the Gait Cycle

We propose a model that can predict the gait percentage that was equally divided into 100 one-percent fragments. To achieve this, we reused the method from [44] to segment input signals and label output targets. We began by extracting the lengths of the walking steps from one heel-contact to the next. Then, we sampled each heel-strike window with an interval of 10 ms. This resulted in several signals that were stored as a matrix *X* of dimension $R^{p\times (s*d)}$, where *p* is the percentage value, *s* is the number of sensors, and *d* is the number of dimensions in an IMU sensor.

### 3.3. Gait Prediction Model

We developed an exponentially delayed fully connected neural network (ED-FNN) that accurately detects and forecasts gait percentage that was densely discretised. In Section 2 we showed that Evans and Arvind [23] implemented a combination of FNN and HMM that had been previously used to detect phases that had been partitioned into five events. However, coarse discretisation of the gait is not sufficient to fully control prosthetic devices for real-world applications. In this section we will describe the ED-FNN and show that this algorithm manages to simulate recurrent neural networks for regression problems.

#### 3.3.1. Fully Connected Neural Networks (FNNs)

An FNN is a collection of artificial neurons called computational units. These units are grouped as a set of layers that are arranged in a hierarchical structure. FNNs are divided into *input layer*, *hidden layers*, and *output layer*. The function of the input layer is to directly process the data given by the user and forward it to the first hidden layer to learn complex representation of the data. This forward process is repeated in the following hidden layers, allowing them to learn more specific characteristics of the input data. The function of the output layer is to process the output of the last hidden layer and generate a prediction that agrees with the ground truth of the given input data. This network architecture is called a *fully connected network* because every unit in a layer is connected to every other unit in the following layer. These connections are represented as weights and biases that express the importance of a respective input to the output. The activation of each unit in a layer of the network is computed by the following equation:

(1) $$a^{l}=\sigma \left(\theta^{l}a^{l-1}+b^{l}\right),$$

where $\theta \in R^{n\times k}$ is a matrix denoting the weights between layers *l* and l−1, $a^{l-1}\in R^{k}$ denotes the activation units of the previous layer (l−1), and σ is a predefined activation function. In the literature, several activation functions (e.g., sigmoid [45], rectified linear unit (ReLU) [46], and softmax [47]) can be found. For our model, we chose a ReLU activation function as shown in Equation (2) due to its properties of avoiding saturation in the error gradients:

(2) σ(z)=max(0,z).

Modifying the weights and biases in every activational layer ($a^{l}$) will lead the overall model to obtain a desirable output. To learn these weights and biases, the objective of the network is quantified by means of a cost function. Several cost functions for neural networks (NN) are found in the literature, such as the mean square error (MSE) [48] and cross-entropy [49]. For the purpose of gait percentage detection, we use the mean square error (MSE) of Equation (3) to minimise the cost between the ground truth of the data and the prediction of our model:

(3) $$\underset{\left(\theta ,b\right)}{MSE}\left(x\right)=\frac{1}{2N}\sum_{x_{i}\in x}^{N}\left|\right|h_{\left(\theta ,b\right)}\left(x_{i}\right)-y\left(x_{i}\right){\left|\right|}^{2},$$

where $h_{\left(\theta ,b\right)}\left(x_{i}\right)$ is the prediction of the FNN and θ and *b* are the weights and the biases of the network. This equation indicates that if the MSE is close to zero, then the weights and the biases reflect a good representation of the given data. In an NN, *gradient-based methods* are used to back-propagate the error from the output layer to every weight of the hidden units. The error gradient indicates the direction in which the weights and biases of the units need to be updated. (Stochastic) gradient descent [50], conjugate gradient [51], and Adam [52] are the most popular gradient-based methods that are used in NNs.

#### 3.3.2. Exponentially Delayed Fully Connected Neural Network (ED-FNN)

**Figure 2:**
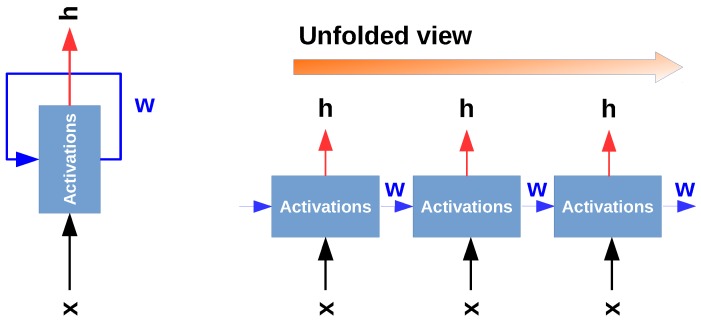
This figure illustrates the information flow in a recurrent neural network (RNN). The left image shows an RNN as an infinite loop network where the model outputs are fed back as inputs. The right figure is an unfolded representation of an RNN [53].

Because FNNs do not hold any notion of time as they only consider the current example *x*, a machine learning model needs to rely not only on the signal taken at time *t* but also on the history of signals $X_{d}=\left[x_{t-k},\cdots ,x_{t}\right]$ to estimate present and future gait percentages $y=[y_{t},y_{t+1},\cdots ,y_{t+n}]$. In this case, *n* indicates the number of gait events to estimate in the future, and *k* specifies the number of IMU samples to take from the past. Recurrent neural networks (RNNs) are known to simulate a historical behaviour by introducing *memory* that encodes information about what has been observed in the past. Figure 2 shows the architecture of an RNN.

In a simple fully connected neural network (FNN), information flows back and forth from the lower to the higher layers of the network, allowing it to learn higher-order representations of the input data. A similar process is observed in RNNs, with the distinction that the network not only depends on the inputs *X*, but also on the activations of the hidden units at previous time steps. As a result, these networks will learn to map the sequence of inputs $x=[x_{t-k},\cdots x_{t}]$ into output of sequences $o=[o_{t+1},\cdots ,o_{t+n}]$. RNN algorithms such as long short-term memory (LSTM) networks have shown great success in many problems that contain temporal information (e.g., IMU signals) [54]. Based on the characteristics of RNNs, it is clear that these networks have the capacity to accurately predict dense gait events. However, we found that they generally require substantially more data than standard FNNs, and they are computationally expensive, which poses limitations when working with microprocessors. Consequently, in our research we created an NN architecture that simulates the “memory” of RNNs and removes the aforementioned limitations.

**Figure 3:**
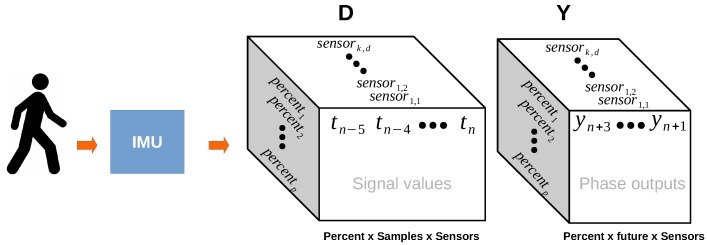
This figure illustrates how the matrix *D* was created. Every sample in the inertial measurement unit (IMU) is delayed by *n* times (in this case five times). The output matrix is shifted *n* times into the future (in this case three times).

To simulate a “memory” of an RNN, the input tensor *X* was delayed according to the following equation:

(4) $$D={\left[W_{(t-d):t}\right]}_{t=d}^{T-1-f},$$

where $W{\left[\cdot \right]}_{start:end}$ is a window sequence from start to end, ${\left[x_{t}\right]}_{A}^{B}$ is a vector of elements $x_{t}$ that ranges from *A* to *B*, *T* indicates the number of samples in the signal, and *f* is the number of percentage values that will be predicted in the future. This equation constructs a tensor *D* of dimensions $R^{p\times s_{d}\times s}$, where *p* indicates percent index, $s_{d}$ indicates the delayed samples, and *s* indicates the number of IMU sensors. This tensor is illustrated in Figure 3. Furthermore, the tensor *D* was reshaped into a matrix $X_{d}\in R^{p\times k}$ in order to use this tensor directly in the NN. In this case, $k=s_{d}*s*d$ refers to the product of the delayed samples, IMU sensors, and the dimensions of the IMU sensors. Finally, we generated the output matrix $Y_{d}\in R^{p\times f}$, where *p* indicates the percentage index and *f* is the number of samples in the future. Using the matrix, we oblige the network to always predict *f* percentage values in advance.

Using the matrix $X_{d}$ directly in an FNN with a *short* delay *d* will yield bad predictions on the overall trend, but it will be good at predicting fast changes in the percentage. This is because a one-to-one mapping between neighbouring samples $X_{d}$ and percent samples $y_{d}$ does not exist. In contrast, using a $X_{d}$ with long delays will be better at predicting the overall trend of the percentage, but less good at predicting fast changes. A good trade-off between large and short delays will yield an optimal input space to predict the gait percentage. One of the reasons why LSTM works well for time series is due to its ability to choose which of the delay samples are important for the overall prediction. As a result, in order to simulate this behaviour, we introduced the concept of *exponential windows*, which allows us to make trade-offs between short and long delays. An exponential window is defined as:

(5) $$\psi \left(X_{d},\delta \right)=X_{d}{\left[t-exp\left(k\right)\right]}_{k=0}^{\delta }.$$

This equation uses the delayed matrix $X_{d}$ and re-samples it using a smaller delay δ. Consequently, we not only obtain a window that includes the knowledge of samples that are close by, but also obtain knowledge of samples that are far away. Note that the number of samples which are close in time are more densely sampled than those that are far away from *t*. This means that we are including information that encompasses both fast changes in the percentage and samples that contribute to the prediction of the cycle trend. Furthermore, applying this *exponential window* allows us to decrease the number of input units that are needed to predict the gait percentages, which results in a substantial decrease of computational power. Additionally, due to the forecasting properties of the network, we also removed the delay limitations that arise when using algorithms on micro-controllers with low memory and CPU power.

**Figure 4:**
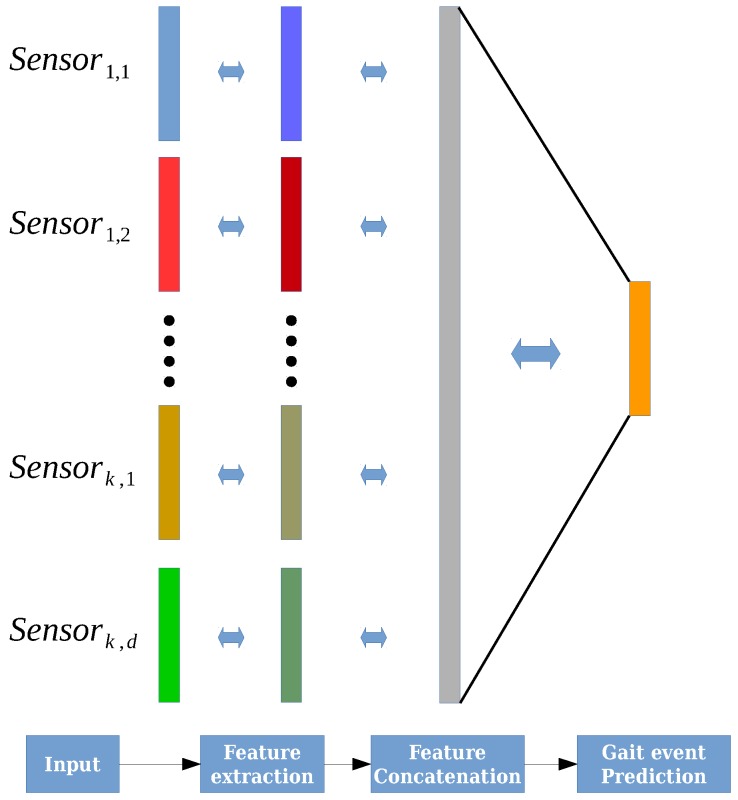
This figure illustrates the exponentially delayed fully connected neural network (ED-FNN) architecture. Initially, the network individually receives each sensor input from the matrix *X* in Equation (5). Then, the network separately extracts the features of each sensor and concatenates them into a single feature vector. Finally, the output layer uses the feature vector to forecast the gait events of the cycle.

The network that was used in our experiments is made of one fully connected (fc) layer as input, two hidden layers of six units, and an output layer of one unit. Furthermore, we found that training one IMU sensor per input layer and concatenating them later decreased the variance between several runs. Figure 4 illustrates the architecture of the NN. The concatenation of the weights and biases for each layer was performed based on the following equation:

(6) $$\begin{array}{cc} a^{lc} & =[a_{0}^{l-1},\cdots ,a_{s}^{l-1}],\\ \theta^{lc} & =[\theta_{0}^{l},\cdots ,\theta_{s}^{l}],\\ b^{lc} & =[b_{0}^{l},\cdots ,b_{s}^{l}], \end{array}$$

where *s* is the number of sensors that were modelled by different FNNs. Note that the activation of the concatenated layer can be computed by modifying Equation (1) as:

(7) $$a^{l}=\sigma \left(\theta^{lc}a^{lc}+b^{lc}\right).$$

Regarding the framework of the ED-FNN, in order to keep the network architecture constrained by the computation complexity, we chose six hidden units per layer. We found that this number of units satisfied the computational limitations and the accuracy of the model. Moreover, with regard to the sampling of Equation (5), we chose an exponential window of 1.6. We found that this window yielded the best results for every subject in the dataset. Further optimisation of the hyper-parameters could be done in future work by means of cross-validation techniques.

#### 3.3.3. Performance Metric for the ED-FNN

To train the ED-FNN model, we used the MSE to minimise the cost between the prediction and the ground truth. In addition to these metrics, we also calculated the mean absolute error (MAE) and the coefficient of determination ($R^{2}$). The absolute error is computed as:

(8) $$MAE=\frac{\sum_{i}\left|{\hat{y}}_{i}-y_{i}\right|}{n},$$

where ${\hat{y}}_{i}$ is the predicted percentage of the model and *y* is the ground truth. Furthermore, we computed $R^{2}$ as:

(9) $$\begin{array}{cc} SS_{tot} & =\sum_{i}{\left({\bar{y}}_{i}-y\right)}^{2},\\ SS_{res} & =\sum_{i}{\left({\hat{y}}_{i}-y\right)}^{2},\\ R^{2} & =1-\frac{SS_{res}}{SS_{tot}}, \end{array}$$

where ${\bar{y}}_{i}$ is the mean of the ground truth and $SS_{tot}$ and $SS_{res}$ indicate the *total sum of squares* and *the residual sum of squares*, respectively. This metric is generally used to measure the correlation between the prediction and the ground truth. If there is a correlation of 1, it means that the prediction fully represents the ground truth.

## 4. Experiments

This experimental section is divided into three parts. First, Section 4.1 explains the electronic board that was used to read the IMU signals. Section 4.2 describes the number of subjects and situations that were performed in the experiments. Finally, Section 4.3 explains how the signals were recorded and pre-processed.

### 4.1. Experimental Electronic Board Prototype, Experiment Protocol, Measurement System

In our experiments, an electronic board with one embedded IMU sensor was used. The board was an Adafruit Feather M0 Bluefruit LE (using ATSAMD21G18 ARM Cortex M0 processor, clocked at 48 MHz and at 3.3 V logic, 256 KB of FLASH ROM and 32 KB of RAM). Moreover, the charging battery unit was designed to measure and monitor the voltage of the battery so we could detect when it needed to be recharged. The board also supports a Bluetooth Low Energy component. This addition makes it convenient for transferring data to the computer or designing mobile applications so that amputees can easily monitor or even control their prosthesis. To measure the gait signals, we used one IMU and two FSRs. The IMU (MPU 6000–Invensense) consists of a gyroscope sensor and an accelerometer. This provides tri-axis signals of angular velocity and tri-axis acceleration of the lower shank. Furthermore, the IMU was connected to the microcontroller via the SPI interface for the purpose of high-speed signal transfer to the commuter (up to 1 MHz). The gyroscope resolution was set at a full range scale of ±2000 degrees/sec with a sensitivity of ±16 g LSB/degree/s. Moreover, the resolution of the accelerometer was set at a full range scale of ±16 g with a sensitivity of 2048 LSB/g (g = 9.8 m/s${}^{2}$). Regarding the FSRs, we placed two of these sensors under the toe and the heel of the subject to detect the impact of the foot with the ground. FSR signals were used as references for classifying gait events and to build a dataset for training the ED-FNN algorithm. After pre-prossessing the IMU signals with the FSRs, we removed these sensors for the training and prediction of the model. All signals were recorded synchronously at intervals of 10 ms, then transmitted directly to the computer. This electronic board was first used for collecting the data and creating a dataset. Additionally, we embedded the gait percent detection algorithm combined with a device control program for the prosthesis.

### 4.2. Subjects

The data were extracted from seven healthy subjects with IMUs fixed with a belt placed on the subjects’ lower shank, and two FSRs were placed under the toe and heel. Participants were five males and two females. Their age ranged from 25 to 33 years, their height ranged from 160 to 185 cm, and their weight ranged from 48 to 80 kg. We recorded different scenarios in two environments. In the first environment, subjects walked on a treadmill with a 0 degree inclination. In the second environment, subjects walked outside on a 0 and a 15-degree inclination. On the treadmill, each subject was required to walk four different trials with different speeds. The speeds were divided into 2.2 m/s, 2.6 m/s, 3.2 m/s, and 3.8 m/s respectively. For outside walking, subjects performed normal speed (approx. 3.2 m/s) and fast speed (approx. 4.0 m/s). All trials were recorded for an interval of five minutes. The number of steps of each subject was categorised as an example in the training data. On average, each participant walked 275.0 steps in the overall experiment. All IMU signals obtained from each participant were mixed together to build a larger dataset for training the network model. This resulted in a dataset of 2313 walking cycles, as shown in Table 2. Merging the dataset allowed the model to increase the chances of extracting the important features that are relevant in different walking gaits.

### 4.3. Off-Line Data Analysis

**Figure 5:**
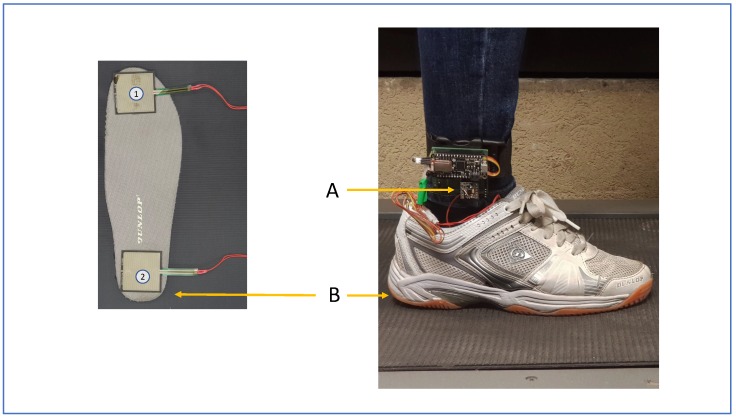
Sensor positions of the IMU and the FSR on the foot. Arrow (A) illustrates the position of the IMU, and arrow (B) the position of FSRs under the sole.

As mentioned earlier, FSRs were used to extract gait cycles and phases by measuring the heel-strike and the toe impact with the ground. The location of FSRs under the sole can be seen in the right image of Figure 5. The IMU’s position on the subject’s lower shank can also be seen in the right image.

**Figure 6:**
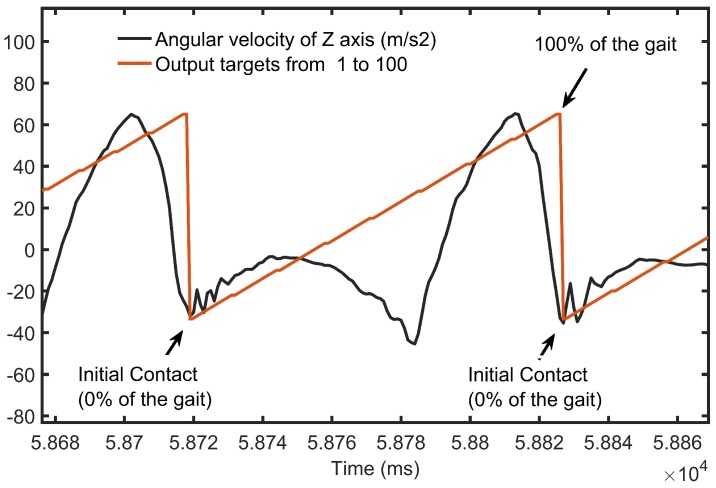
The figure shows one gait cycle discretised with a 1% interval. The division was based on measuring cycle latency, from an initial-contact (IC) at 0% to the next at 100%.

In Section 2 we described that the methodologies that divide the gait cycle into foutr or more phases (see Table 1) yield acceptable results to classify the gait percentage. However, for real-world applications, this phase granularity may not be sufficient for controlling active prosthetics. For this reason, we want to open an opportunity for future active prosthetics to be able to fully control their devices by accurately predicting the gait percentage in a densely discretised gait. An example can be observed in Figure 6, which illustrates a gait cycle that was discretised within a 1% interval in the real-time estimation of the gait cycle. Lastly, in comparison with [3,34,37,55], our model does not require complex pre-processing steps, as it can deal with data with a high signal-to-noise ratio.

## 5. Results

The development of a gait percent detection model based on ED-FNN was described above. The model was trained with the angular velocity and acceleration signals in the sagittal plane of the foot. These signals were taken from healthy subjects walking at different speeds on flat ground and on a 15-degree inclined terrain. The performance of our method was evaluated on individual and group bases. Over several runs, we computed the mean loss and variance to determine the overall performance of the model. Additionally, we generated a validation set to validate the generalisation accuracy of our model.

**Figure 7:**
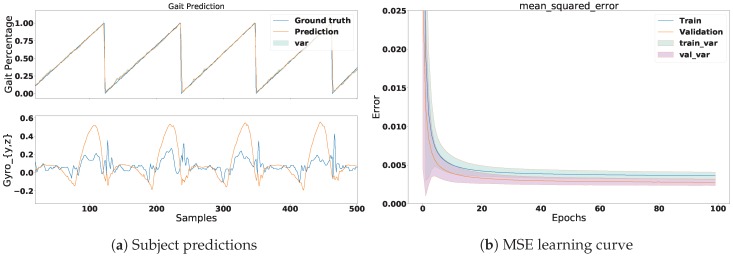
This figure shows the prediction and the results of the learning process for one subject. (**a**) The ground truth and mean prediction of the gait phase discretisation divided into 100 portions normalised between 0 and 1 (0 equals to 0 percent and 1 equals 100 percent of the gait cycle). The bottom figure shows the *y* and *z* signals of the gyroscope sensor; (**b**) The mean and variance of the mean square error (MSE) learning curve. The average of MSE reached a loss of 0.003 in the training set and 0.0662 in the validation set.

**Figure 8:**
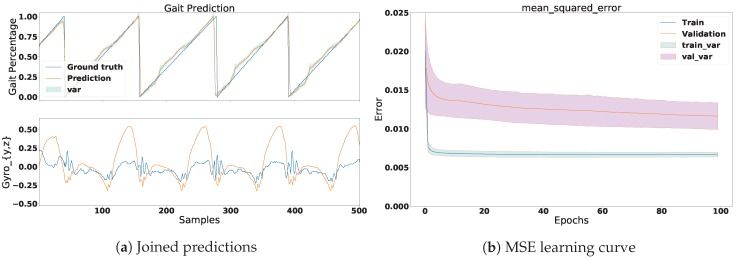
This figure shows the prediction and learning process results of the joined signal for several subjects. Similar to Figure 7, (**a**) shows the comparison between the prediction and the ground truth and (**b**) illustrates the learning curve of the MSE. The average MSE reached a loss of 0.006 in the training set and an average of 0.0115 in the test set.

**Figure 9:**
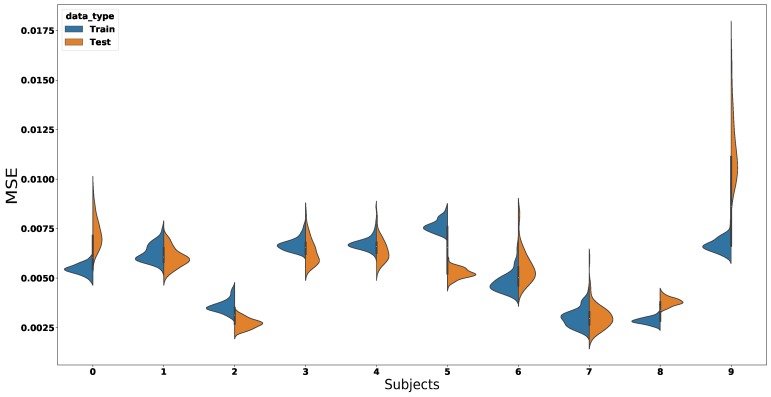
This figure illustrates the MSE for every subject in the experiments. Each number in the *x*-axis from 1 to 7 represents a subject in the experiments. Violin plots 7, 8, and 9 were of two 15 degree incline walks and to all subjects’ signals combined, respectively. Every violin plot consists of two distributions (i.e., Train—blue and Test—orange ) and the mean of the MSE. The distributions illustrate the MSE variance over 100 runs. This figure shows that the ED-FNN managed to accurately predict the gait cycle over several subjects.

For each subject, we computed the mean absolute error (MAE), mean square error (MSE), and the coefficient of determination ($R^{2}$). Table 3 gives an overview of the average error of all subjects and the joined dataset. These values are shown as training and validation errors. Furthermore, due to the number of subjects in the experiments, the visualisation of the results was divided into two parts. The first part illustrates the learning process of the MSE prediction in four different plots: two for one single subject and two for all-subjects’ signals combined. The second part individually illustrates the overall MSE performance for every subject by means of a violin plot. The following table provides a summary of the performance in Figure 7, Figure 8 and Figure 9.

(10) $$MSE_{\%}=\sqrt{MSE}\times 100$$

(11) $$MAE_{\%}=MAE\times 100$$

### 5.1. Results: Part 1

In this section we illustrate the results for one subject and all the subjects’ signals combined. Figure 7 shows the prediction and learning process of one subject. The $Gyroscope_{y,z}$ signals can be observed in the bottom plot in Figure 7a. Additionally, the prediction of the phase signal can be observed in the top plot. Notice that the prediction almost perfectly follows the trend of the ground truth. Moreover, we can see that this accuracy is reflected in Figure 7b, which shows the MSE of the learning. In this plot we can observe that the MSE reached an average loss of 0.003 in the training set and a value of 0.0028 for the validation set. The reason why the model performed better in the validation set is because it was slightly under-fitting the data. This could be solved by increasing the size of the NN. Figure 8a illustrates the gait prediction for all subjects’ signals joined together. In this plot we can observe similar results to those in Figure 7a, with a difference in the accuracy of the MSE. Here we can see that the validation set performed less well than in one subject alone. This was expected, as the signals of each subject slightly vary. Furthermore, we can observe that the prediction still followed the trend of the ground truth despite the decrease of accuracy. Based on these results, we can conclude that the network managed to generalise well.

### 5.2. Results: Part 2

This section describes the MSE violin plot for every individual subject in the experiments. In Figure 9, the colours of the plot show the distributions of the training and the test MSEs. Each violin in the plot belongs to one subject in the experiments, and the last violin plot belongs to all the subjects’ signals combined. These results show that the ED-FNN accuracy was consistent over every subject in the dataset. Furthermore, thanks to the concatenation of the layers in our network, we can observe that the variance between different runs was small. As a result, it increased the robustness and reliability of our model.

### 5.3. Reference System

We observed that our algorithm was able to accurately predict 100 percent of the gait cycle. To our best knowledge, this setting has never been done in previous studies. Furthermore, we showed that the MSE managed on average to achieve a 0.003 in both validation and training sets. We expect the real-time performance to be very similar to the off-line performance. It is difficult to compare our algorithm with other studies, as our setting is not standard. However, Table 4 lays out the performance of other existing gait phase prediction systems using one IMU.

## 6. Conclusions and Future Work

Over the recent decades, gait phase detection algorithms have become a challenging topic for researchers due to their extensive application in assistance devices. One example is improving the gait phase detection accuracy in prosthetics so that amputees can safely use these devices. To date, many gait phase detection methods have been developed. However, these methods use only one sensor, and often detect gait cycles on a low granular domain of the phase space. In order to take full control of current prosthetics, we proposed a robust walking gait percent detection method that can detect 100 percent of the gait cycle for walking on flat ground and on a 15-degree inclination. Because other similar methods have shown that the accuracy of gait phase detection algorithms is suitable for ambulatory applications, we showed that our method yielded state-of-the-art performance for these applications. In summary, our study obtained the following outcomes:

- A compact system using one IMU mounted on the lower shank.
- A model that is capable of learning highly discretised percentages of the gait cycles.
- An average mean square error of approximately 0.003 in both training and validation sets for single subjects.
- A model that generalises toward several subjects with an average MSE of 0.006 in the training set and 0.01 in the validation set.
- A model that is consistent over several subjects. (i.e., low variance between several runs).
- A model with powerful forecast capabilities that introduces a no-delay prediction method within 10 ms.

It is important to note that our tests were performed on ARM chips, which are known to underperform when conducting heavy mathematical computations. Because our experiments were purely offline, computational cost was not a concern. However, our model was built with the purpose of working on scenarios where computational power is an issue. The ED-FNN requires less samples than a normal FNN or RNN. Consequently, it takes less computational power for prediction. Additionally, we also included a forecasting option that allows the network to predict future percentages in the case of a delay in the system. Furthermore, we would like to point out that we are not learning on the ARM chips directly. Instead, we learn the models in a normal computer and then make the predictions in the ARM chip. Lastly, in the near future we plan to leave aside the Beagle Bone Black and use other more powerful alternatives, such as ODROID-C2 boards or neuromorphic computation.

For future work we will focus on improving the prediction accuracy and evaluation with different walking conditions such as stair walking and real-time implementation with the newest prosthesis version of the AMP-Foot [4]. Additionally, the hyper-parameters of the ED-FNN model will be improved by means of cross-validation methods.

All subjects gave their informed consent for inclusion before they participated in the study. The study was conducted in accordance with the Declaration of Helsinki, and the protocol was approved by the Ethics Committee of B.U.N 143201526629.

## Figures and Tables

**Table 1:** One gait cycle can be subdivided into eight typical phases based on the gait fundamentals shown in Section 3.1. In this table *Dorsi Assist* means assisting the foot to bend up, *Plantar Assist* means assisting the foot to bend down, and *No Assist* means that there is no assistant for the movement. FF: foot-flat.

Label	Phase	Percentage	Function	Controlling
0	Initial Contact	0 to 8	Loading, weight transfer	Dorsi Assist
1	Mid Mid-stance (FF)	8 to 30	Support of entire body weight:	No Assist
2	Terminal Mid-stance (FF)	30 to 40	Center of mass moving forward	No Assist
3	Push Off	40 to 50	Push Off	Plantar Assist
4	Pre-swing, double-limb support, push off	50 to 60	Unloading and preparing for swing	Plantar Assist
5	Initial swing	60 to 75	Foot Clearance	Dorsi Assist
6	Midswing	75 to 85	Limb advances in front of body	Dorsi Assist
7	Terminal Swing	85 to 100	Preparation for weight transfer	Dorsi Assist

**Table 2:** The number of samples and cycles in the dataset.

Subjects	The Number of Samples	The Number of Cycles
Subject 1	19,805	162
Subject 2	47,089	449
Subject 3	46,367	434
Subject 4	21,531	189
Subject 5	19,149	170
Subject 6	15,858	181
Subject 7	25,166	258
Subject 8	15,858	181
Data on the treadmill	78,473	451
Dataset (all samples and cycles)	269,491	2313

**Table 3:** The average error across every subject in the dataset and the error obtained when learning in the joined dataset. To convert these errors into percentages (except $R^{2}$), the equations for MSE and MAE are given in Equations (10) and (11), respectively. Individual errors for each subject are shown in Figure 9.

	Error					
	t-MSE	v-MSE	t-MAE	v-MAE	t-$R^{2}$	v-$R^{2}$
**Average**	0.005±0.0003	0.005±0.0004	0.021±0.001	0.023±0.002	0.93±0.003	0.881±0.015
**Joined**	0.006±0.0003	0.011±0.0017	0.021±0.001	0.041±0.002	0.91±0.004	0.828±0.022

**Table 4:** This table compares existing gait event predictions with our method using one IMU. Every method was applied on lower limbs. FF: foot-flat; HO: heel-off; IC: initial contact; TO: toe-off.

Author	Detectable Events or Phases	Performance	Metric	Detection
Ledoux et al. [42] (2018)	IC and TO	−1.7%±0.6 stride (IC), −1.8%±0.6 stride (TO)	Detection delays	On-line
Zakria et al. [12] (2017)	IC and TO	3.92 ms ± 1.56 (IC), −1.81 ms ± 4.03 (TO)	Time difference	Off-line
Maqbool et al. [41] (2016)	IC and TO	15.44 ms ±25.2 (IC), −28.44 ms ±16.2 (TO)	Time difference	On-line
Zhou et al. [9] (2016)	IC and TO	95% (TO: upstairs), 99% (IC: upstairs), 99% (TO: downstairs) 98% (IC: downstairs)	Detection precision	On-line
Mannini et al. [17] (2014)	IC, FF, HO, TO	62 ms ± 47 (IC), −3 ms ± 53 (FF), 86 ms ± 61 (HO), 36 ms ± 18 (IC),	Time difference	On-line
Muller et al. [39] (2015)	Detected four phases	100 ms ± 50 (TO), 50 ms ± 79 (IC)	Time difference	On-line
Quintero et al. [40] (2017)	100 gait percent	Reported visually	Theory	Off-line
Our method	100 gait percent	2.1%± 0.1	MAE—No delay	Off-line
